# Predicting Survival from Telomere Length versus Conventional Predictors: A Multinational Population-Based Cohort Study

**DOI:** 10.1371/journal.pone.0152486

**Published:** 2016-04-06

**Authors:** Dana A. Glei, Noreen Goldman, Rosa Ana Risques, David H. Rehkopf, William H. Dow, Luis Rosero-Bixby, Maxine Weinstein

**Affiliations:** 1 Center for Population and Health, Georgetown University, Washington, District of Columbia, United States of America; 2 Office of Population Research, Princeton University, Princeton, New Jersey, United States of America; 3 Department of Pathology, University of Washington, Seattle, Washington, United States of America; 4 Division of General Medical Disciplines, School of Medicine, Stanford University, Stanford, California, United States of America; 5 School of Public Health, University of California-Berkeley, Berkeley, California, United States of America; 6 Centro Centroamericano de Población, Universidad de Costa Rica, San Jose, Costa Rica; Innsbruck Medical University, AUSTRIA

## Abstract

Telomere length has generated substantial interest as a potential predictor of aging-related diseases and mortality. Some studies have reported significant associations, but few have tested its ability to discriminate between decedents and survivors compared with a broad range of well-established predictors that include both biomarkers and commonly collected self-reported data. Our aim here was to quantify the prognostic value of leukocyte telomere length relative to age, sex, and 19 other variables for predicting five-year mortality among older persons in three countries. We used data from nationally representative surveys in Costa Rica (*N =* 923, aged 61+), Taiwan (*N =* 976, aged 54+), and the U.S. (*N =* 2672, aged 60+). Our study used a prospective cohort design with all-cause mortality during five years post-exam as the outcome. We fit Cox hazards models separately by country, and assessed the discriminatory ability of each predictor. Age was, by far, the single best predictor of all-cause mortality, whereas leukocyte telomere length was only somewhat better than random chance in terms of discriminating between decedents and survivors. After adjustment for age and sex, telomere length ranked between 15th and 17th (out of 20), and its incremental contribution was small; nine self-reported variables (e.g., mobility, global self-assessed health status, limitations with activities of daily living, smoking status), a cognitive assessment, and three biological markers (C-reactive protein, serum creatinine, and glycosylated hemoglobin) were more powerful predictors of mortality in all three countries. Results were similar for cause-specific models (i.e., mortality from cardiovascular disease, cancer, and all other causes combined). Leukocyte telomere length had a statistically discernible, but weak, association with mortality, but it did not predict survival as well as age or many other self-reported variables. Although telomere length may eventually help scientists understand aging, more powerful and more easily obtained tools are available for predicting survival.

## Introduction

Human telomeres shorten with age in leukocytes as well as in other tissues [1]. Thus, telomere length has generated substantial interest as a potential predictor of age-related diseases and mortality. A number of studies that examined the association between leukocyte telomere length (LTL) and all-cause mortality found a statistically discernible relationship [2–14], but few studies explicitly compared LTL with other predictors of mortality: three [9, 15, 16] compared LTL with other biomarkers, and one [17] compared LTL with both biomarker and other predictors. These four studies [9, 15–17] focused on effect sizes and/or the significance of LTL; none quantified the discriminatory ability of LTL and compared it with a range of established mortality predictors such as those included in existing prognostic indexes [18]. A further limitation of these four studies is that they were based on samples of very old individuals and in one case [17] drew from a clinical population of discharged hospital patients. Unlike this investigation, none was based on a nationally-representative sample including cohorts young enough to have only minimal bias from selective mortality.

Telomeres—the repetitive DNA sequences that cap the chromosomes to protect them from fusion and degradation—shorten with each cell division [19]. Eventually, they reach a critical minimum length, triggering the cell to stop dividing [20]. Thus at the cellular level, telomeres act as a ‘molecular clock’, but to what extent they explain organismal aging—or mortality—is debatable [21, 22]. Some suggest that cell senescence triggered by telomere dysfunction contributes to the decline in tissue function we associate with aging [23]. Indirect evidence supports this view: some genetic diseases associated with premature aging also lead to telomere shortening [24, 25], and genetically modified mice with short telomeres manifest symptoms reminiscent of human aging [24]. Genetic variance studies have also identified several genetic markers that are associated with both telomere length and various age-related diseases or mortality [26, 27]. Thus, LTL has gained popularity as a marker of aging. Yet, a rigorous comparison of the ability of LTL versus well-established predictors to discriminate decedents from survivors is lacking.

Our study focuses on all-cause mortality. Mortality is an attractive metric of aging because death is a well-defined and salient outcome with minimal measurement error when vital status is determined from virtually complete death registration records. Prior evidence regarding the relationship between LTL and mortality has been mixed. Telomere length was found to be longer in a high longevity region of Costa Rica as compared to the rest of the country [28]. While some studies have reported an inverse association between LTL and all-cause mortality [2–14]; others have found no relationship [15–17, 29–37] or only a marginally significant association [38, 39]. Recently, the largest study to date [8] reported an association between short LTL and mortality, including cancer mortality, although genetically-determined short telomeres were protective of cancer mortality. Thus, the relationship between shorter telomeres and mortality appears to be a complex one, potentially confounded by multiple factors, and causal linkages have not been established [7]. Here we pose a more fundamental question: can LTL predict mortality better than other well-established and less costly predictors?

Many studies of LTL and mortality focus on the significance of the association, but statistical significance is not a sufficient criterion to evaluate the incremental value of a marker. Similarly, the effect size or magnitude of the association (e.g., a hazard ratio) is useful for identifying risk factors, but it is not an appropriate statistical tool for quantifying predictive accuracy because even strong associations (e.g., large hazard ratios) may yield little improvement in discrimination [40, 41]. For example, a large and significant hazard ratio associated with extreme values of a particular biomarker may not distinguish well between survivors and decedents in a statistical model if very few people have such extreme values of the biomarker. In contrast to previous approaches, here we quantify the prognostic or discriminatory value of LTL for predicting five-year all-cause mortality in terms of its ability to differentiate between decedents and survivors compared with 21 well-established predictors of mortality. We use data from nationally representative samples of older persons in Costa Rica (ages 61+), Taiwan (ages 54+), and the U.S. (ages 60+).

## Materials and Methods

### Data

Data come from the second wave (fielded in 2006–08) of the Costa Rican Study on Longevity and Healthy Aging (CRELES), the 1999–2002 waves of the National Health and Nutrition Examination Survey (NHANES), and the 2000 wave of the Social Environment and Biomarkers of Aging Study (SEBAS). Details regarding sampling design and response rates for each dataset are provided elsewhere [42–44].

Among 2364 respondents aged 61 and older who completed the CRELES wave 2 interview, 2166 provided a blood sample from which DNA was extracted and banked. A subsample (*N =* 994) of these respondents was selected for the LTL assay, including all those from the Nicoya region and a probability sample of the remainder. The concentration of the stored DNA specimen was insufficient for 71 of those selected, leaving an analysis sample of 923.

For NHANES, we restricted the analysis to persons aged 60 and older (for comparability with the other samples and to allow for the inclusion of cognitive function, which was not asked of respondents younger than 60), among whom 3706 completed the interview, 3234 participated in the exam, and 3068 were eligible for blood sampling. Of those eligible, the analysis sample comprised 2672 individuals who supplied a DNA specimen.

Among 1497 respondents aged 54 and older who completed the 2000 SEBAS household interview, 1386 were eligible for the exam and 111 were ineligible because of a health condition. Of those eligible, 363 refused the exam and 47 had insufficient DNA, leaving an analysis sample of 976.

### Ethics Statements

All three surveys obtained written, informed consent from all participants and received human subjects approval from the institutional review boards (IRB) at the institutions conducting the studies: the Ethical Science Committee of the University of Costa Rica (VI-763-CEC-23-04) [CRELES]; Princeton University IRB (#1848, #2193, #2791, #3391), Georgetown University IRB (#1999–195), and the Joint IRB in Taiwan (NIFP-IRB-2000-01) [SEBAS]; NCHS Research Ethics Review Board (Protocol #98–12) [NHANES].

### Mortality

Survival status was determined based on administrative records and in the case of CRELES, complementary survey follow-up (see S1 Appendix). The number of respondents who died within five years was 276 in CRELES, 442 in NHANES, and 128 in SEBAS.

### Predictors

LTL was measured using quantitative polymerase chain reaction (Q-PCR) to determine the relative ratio of telomere to a single-copy gene (T/S ratio) in all three studies, although there were some differences in the assay protocol (see S1 Appendix). The three datasets were analyzed independently (e.g., LTL values from the three studies were not pooled). The inter-assay coefficients of variation for the three studies were: 3.7% for CRELES, 6.5% for NHANES, and 7% for SEBAS.

Within each study, we tested LTL against a broad set of well-established predictors of mortality, many of which are used in existing prognostic indexes (eprognosis.ucsf.edu). They comprise two demographic variables (age, sex), three social factors (marital status, education, and social integration), two health behaviors (smoking, physical activity), six self-reported measures of health status (global self-assessed health, activities of daily living (ADL) limitations, mobility limitations, history of diabetes, history of cancer, and number of hospital days/stays in the past 12 months), a cognitive assessment, and seven biomarkers (systolic and diastolic blood pressure, total cholesterol, glycosylated hemoglobin, body mass index, C-reactive protein, and serum creatinine) in addition to LTL. See Table 1 for details.

**Table 1:** Potential predictors included in the analysis.

	Costa Rica [CRELES]	Taiwan [SEBAS]	U.S. [NHANES]
**Demographic characteristics**			
1) Age at exam	61+	54+	60+
2) Sex	Male, Female		
**Social factors**			
3) Marital status	Categorical: Married/partner; Widowed; Divorced/separated; Never married		
4) Education^a^	Completed Years	Completed Years	Categorical
Very low	None	None	Less than 9th grade
Low	1–2 years	1–5 years	9th– 11th grade
Medium	3–5 years	6 years	High school diploma/GED
High	6–9 years	7–11 years	Some college or associate degree
Very high	10+ years	12+ years	College graduate or higher
5) Social integration index^b^	Based on 5 items; Cronbach’s α = 0.58	Based on 11 items; Cronbach’s α = 0.73	Based on 3 items; Cronbach’s α = 0.72
**Health behaviors**			
6) Smoking status	Categorical: Never; Former Smoker; Current Smoker		
7) Exercise frequency	Dummy: Exercised 3+ times per week in past 12 months	Categorical: None; <3 times per week; 3–5 times per week; 6+ times per week	Cumulative frequency of moderate/vigorous leisure-time physical activities in past 30 days, recoded: None; <12, 12–29, 30+
**Health status (self-reported)**			
8) Self-assessed health status	Based on a simple question that is typically worded: “How would you rate your overall health?” and has five response categories ranging from “poor” to “excellent.”		
9) Number of ADL limitations^c^	Based on 5 ADLs	Based on 6 ADLs	Based on 5 ADLs
10) Index of mobility limitations^d^	Based on 4 physical tasks; coded on a 3-point scale from no difficulty to unable	Based on 8 physical tasks; coded on a 4-point scale from no difficulty to unable	Based on 8 physical tasks; coded on a 4-point scale from no difficulty to unable
11) History of diabetes	Doctor told you that you have…	Ever had…	Ever told by a doctor…
12) History of cancer	Doctor told you that you have…	Ever had…	Ever told by a doctor…
13) Hospital days/stays, past 12 months	Days	Stays	Stays
**Interviewer-administered assessment**			
14) Cognitive function^e^	Based on tasks from the MMSE [45] (basic orientation, immediate & delayed recall, follow a 3-stage command, copy a geometric design) as well as a reverse recall task	Based on several items from the SPMSQ [46] (basic orientation, serial subtraction), a word recall task from the modified RAVLT [47], & a modified version of the Digits Backwards Test [48]	WAIS III (Wechsler Adult Intelligence Scale, Third Edition) Digit Symbol Substitution Test [49, 50]
**Biomarkers**			
15) Systolic blood pressure (SBP)	Mean of 1st & 2nd readings taken at home	Mean of 1st & 2nd readings taken in the hospital	Mean of 1st & 2nd readings taken in the Mobile Exam Center (MEC)
16) Diastolic blood pressure (DBP)	Mean of 1st & 2nd readings (at home)	Mean of 1st & 2nd readings (in hospital)	Mean of 1st & 2nd readings (MEC)
17) Total cholesterol (TC)	Metabolic risk factor associated with cardiovascular disease		
18) Glycosylated hemoglobin (HbA1c)	Marker of glucose metabolism		
19) Body mass index (BMI)	Measure of body fat computed as body weight (in kg) divided by height (in m) squared		
20) C-reactive protein (CRP)	Inflammatory marker		
21) Serum creatinine (SCr)	Marker of renal function		
22) Leukocyte telomere length (LTL)	Measured by the relative ratio of telomere to a single-copy gene (T/S ratio)		

Abbreviations: ADL, Activities of Daily Living; MMSE, Mini-Mental State Exam; RAVLT, Rey Auditory Verbal Learning Test; SPMSQ, Short Portable Mental Status Questionnaire.

^a^ Because education was coded in five categories for NHANES, we recoded education (completed years) into categories for CRELES and SEBAS as well.

^b^ See S1 Table for a detailed list of the components included in the social integration index for each country.

Because the level of measurement varied across items, we standardized each of the components (based on the within-country distribution) and then calculated the mean across valid items if at least 75% items were valid.

^c^ ADLs included: eating; getting out of bed; moving around the house; bathing (CRELES, SEBAS); dressing (NHANES, SEBAS); standing up from a chair (NHANES); using the toilet (CRELES, SEBAS).

^d^ The index was based on difficulty performing various physical tasks without assistance.

Three of the tasks were asked in all three surveys (walking, climbing stairs, reaching overhead), although CRELES asked respondents to demonstrate whether they could lift their arms above their shoulders whereas the other two surveys relied on self-report. The fourth task differed across surveys: pushing/pulling large objects (CRELES), sitting for a long period (NHANES), and running a short distance (SEBAS). NHANES and SEBAS included four additional tasks that were not asked in CRELES: standing for an extended period, lifting or carrying something somewhat heavy, grasping an object with her/his fingers, and bending/kneeling/squatting. Based on the recommendations of Long & Pavalko [51], we constructed the index by summing the available items (potential range: 0–8 in CRELES; 0–24 in NHANES and SEBAS), adding a constant (0.5), and taking the logarithm of the result, which allows for relative rather than absolute effects.

^e^ The summary measure of cognitive function is based on the respondent’s ability to perform various tasks administered by the interviewer.

### Statistical Analysis

A substantial portion of each sample was missing data for at least one predictor. To maximize use of the data, we followed standard practices of multiple imputation (see S1 Appendix). Descriptive statistics were weighted to account for oversampling and differential response rates (S2 Table). All models were fitted separately by country using a Cox hazards model with unweighted data. To quantify the predictive ability of each variable, we used the Area Under the Receiver Operating Characteristic Curve (AUC), a commonly used measure of discrimination with values ranging from 0 to 1, where 0.5 indicates the model performs no better than chance and 1.0 represents perfect accuracy. The AUC can be interpreted as the probability that the model predicts a higher probability of death for those who died than for those who survived [52]. Pencina et al. [40] suggest that an increase of 0.01 in the AUC is a meaningful improvement.

We first tested each predictor individually using duration of follow-up as the metric for time. Education, exercise frequency, and self-assessed health status were treated as categorical in order to allow for non-linear effects. We tested each predictor for non-proportional hazards (i.e., effect of the predictor varies with duration of follow-up). In cases where the interaction between the predictor and duration was significant (*p*<0.05), we included that interaction in the model. In CRELES, the effects of marital status, exercise frequency, systolic blood pressure, and C-reactive protein diminished with time. For NHANES, the effect of diabetes weakened over time. There was no evidence of non-proportional hazards in SEBAS.

Next, we ran models that controlled for age (i.e., using age as the time metric so as to estimate age-specific mortality) and tested each of the remaining 21 predictors individually. To allow for non-proportional hazards (i.e., effect of the predictor varies across age), we tested an interaction between each predictor and age. We included in the final model interactions that were significant: sex, social integration, C-reactive protein, and serum creatinine for SEBAS; marital status, social integration, and exercise frequency for NHANES; but none for CRELES.

Our final models controlled for both age (as the clock) and sex. These models tested the incremental contribution for each of the remaining 20 predictors individually. Again, we included the interactions with age noted above.

## Results

**Fig 1:**
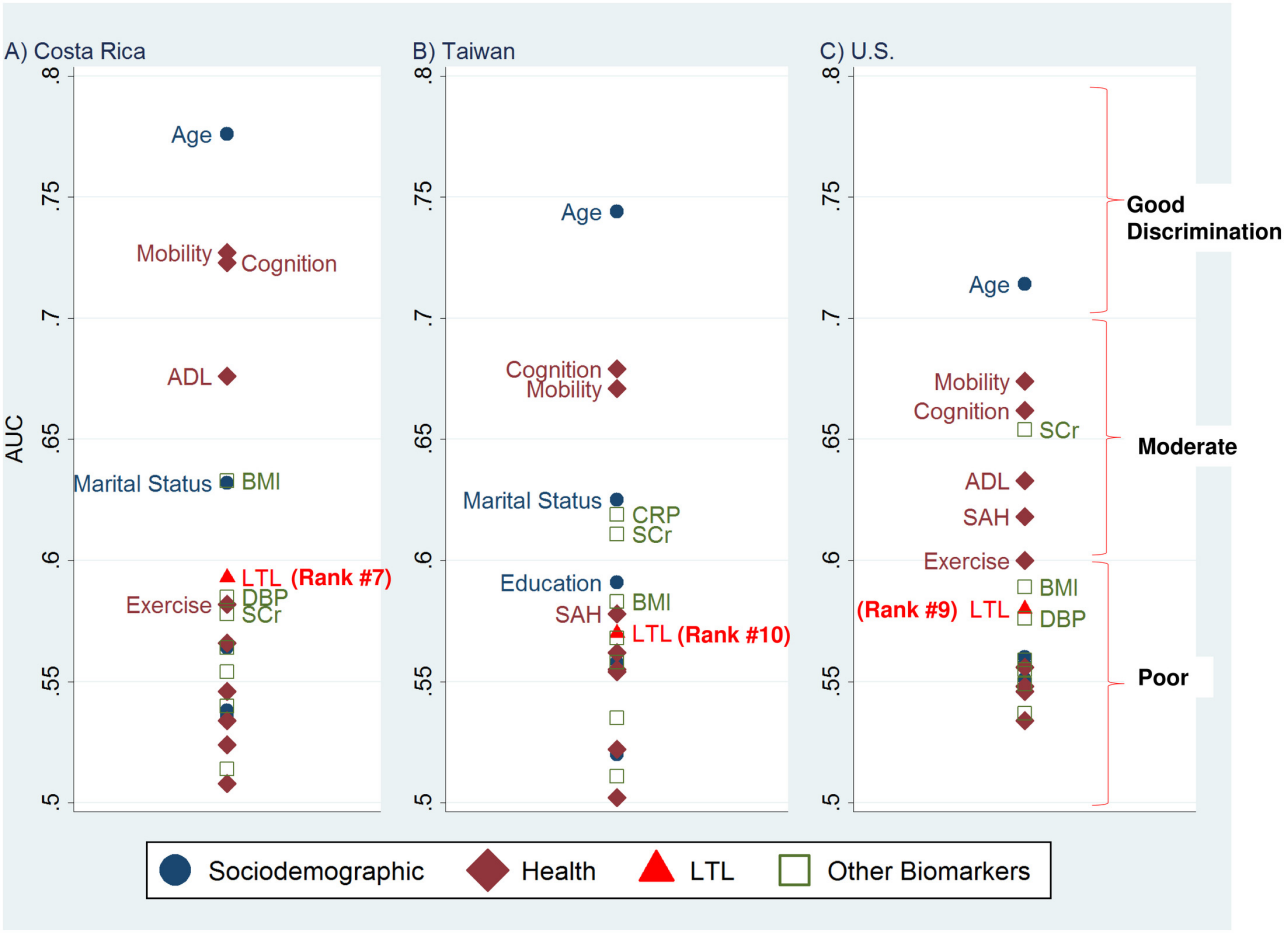
AUC for 22 Potential Predictors of Five-Year Mortality. (A) Costa Rica. (B) Taiwan. (C) U.S. Only the top 10 predictors are labeled. Abbreviations: ADL, Activities of daily living; AUC, Area under the receiver-operating-characteristic curve; BMI, Body Mass Index; CRP, C-reactive protein; DBP, Diastolic blood pressure; LTL, Leukocyte telomere length; SAH, Self-assessed health status; SCr, Serum creatinine.

When the 22 variables were tested individually, chronological age was, by far, the single best predictor of five-year mortality (AUC = 0.78 in Costa Rica, 0.74 in Taiwan, and 0.71 in the U.S.; Table 2). Although LTL was significantly associated with mortality in all three countries (Table 3, Model 1), LTL ranked 7th in Costa Rica, 10th in Taiwan, and 9th in the U.S. (Fig 1). LTL was somewhat better than random chance in discriminating between decedents and survivors: AUC = 0.59 in Costa Rica; 0.57 in Taiwan; 0.58 in the U.S. (Table 2). In addition to age, several self-reported variables and the cognitive assessment were stronger predictors of mortality than LTL. Among the eight biomarkers, LTL ranked second in Costa Rica, fourth in Taiwan, and third in the U.S. Body mass index (inversely associated with mortality) ranked higher than LTL in all countries, while serum creatinine outperformed LTL in two of the three countries. With the exception of LTL, the biomarkers tested here represent clinical markers commonly used in treatment decisions. Yet, five of the eight biomarkers offered weak discrimination (AUC<0.60) in all three countries.

**Table 2:** AUC and Rank for 22 Potential Predictors of All-Cause Mortality, by Country.

	Costa Rica (*N =* 923)		Taiwan (*N =* 976)		U.S. (*N =* 2672)	
Predictor	AUC	Rank	AUC	Rank	AUC	Rank
Age	0.776	1	0.744	1	0.714	1
Sex	0.536	18	0.520	20	0.557	14
Marital status	0.632	6	0.625	4	0.551	17
Education	0.564	13	0.591	7	0.56	11
Social integration	0.538	17	0.558	14	0.557	13
Smoking status	0.566	11	0.555	16	0.548	19
Exercise frequency	0.582	9	0.522	19	0.600	7
Self-assessed health status	0.534	19	0.578	9	0.618	6
Number of ADL limitations	0.676	4	0.555	15	0.633	5
Index of mobility limitations	0.727	2	0.671	3	0.674	2
History of diabetes	0.524	20	0.562	12	0.534	22
History of cancer	0.508	22	0.502	22	0.546	20
Number of hospital days/stays	0.546	15	0.554	17	0.556	15
Cognitive function	0.723	3	0.679	2	0.662	3
Systolic blood pressure	0.564	12	0.558	13	0.555	16
Diastolic blood pressure	0.585	8	0.511	21	0.576	10
Total cholesterol	0.540	16	0.568	11	0.549	18
Glycosylated hemoglobin	0.514	21	0.535	18	0.537	21
Body mass index	0.633	5	0.583	8	0.589	8
C-reactive protein	0.554	14	0.619	5	0.559	12
Serum creatinine	0.578	10	0.611	6	0.654	4
Leukocyte telomere length	0.593	7	0.570	10	0.580	9

**Table 3:** Hazard ratios (HR) and gain in AUC attributable to LTL and selected best predictors of all-cause mortality. CRELES (Costa Rica, N = 923), SEBAS (Taiwan, N = 976), and NHANES (U.S., N = 2672).

	HR^a^			Gain in AUC^b^		
	CRELES	SEBAS	NHANES	CRELES	SEBAS	NHANES
**Model 1: Unadjusted**				*vs*. *Random Chance (AUC = 0*.*50)*		
a) LTL	0.77***	0.82*	0.70***	0.093	0.070	0.080
b) Age	2.40***	2.41***	4.94***	**0.276**	**0.244**	**0.214**
c) Self-Reported Mobility	2.13***	1.79***	1.71***	0.227	0.171	0.174
d) Cognitive Function	0.65***	0.59***	0.63***	0.223	0.179	0.162
e) ADL limitations	1.76***	1.25***	1.39***	0.176	0.055	0.133
**Model 2: Adjusted for age**				*vs*. *Age only*		
a) LTL	1.05	0.93	0.85*	<0.001	<0.001	0.006
b) Self-Reported Mobility	1.45***	1.35**	1.53***	**0.017**	0.013	0.036
c) Self-Assessed Health (SAH)				0.004	**0.020**	**0.042**
Poor (ref)	1.00	1.00	1.00			
Fair	1.06	0.34**	0.42***			
Good	0.90	0.37**	0.30***			
Very Good	0.61+	0.31**	0.21***			
Excellent	0.89	0.17***	0.19***			
d) Cognitive Function	0.84*	0.73***	0.66***	0.008	0.016	0.026
**Model 3: Adjusted for age and sex**				*vs*. *Age and Sex only*		
a) LTL	1.07	0.92	0.88+	0.001	<0.001	0.002
b) Self-Reported Mobility	1.51***	1.45***	1.60***	**0.021**	0.014	0.038
c) Self-Assessed Health Status				0.004	**0.018**	**0.039**
Poor (ref)	1.00	1.00	1.00			
Fair	1.04	0.34**	0.42***			
Good	0.89	0.36**	0.31***			
Very Good	0.61+	0.31**	0.21***			
Excellent	0.89	0.16***	0.19***			
d) Cognitive Function	0.84*	0.71***	0.67***	0.008	0.015	0.021
e) ADL limitations	1.30***	1.12*	1.30***	0.012	0.007	0.026
f) CRP	1.23***	1.42***^,^ ^c^	1.09***	0.010	0.017	0.007
CRP x Age	--	0.99+	--			
**Model 4: Adjusted for all sociodemographic controls**^d^				*vs*. *Sociodemographic controls only*		
a) LTL	1.06	0.94	0.88+	<0.001	<0.001	0.002
b) Self-Reported Mobility	1.48***	1.41**	1.57***	**0.018**	0.011	**0.032**
c) Self-Assessed Health Status				0.005	0.012	0.031
Poor (ref)	1.00	1.00	1.00			
Fair	1.04	0.38**	0.43***			
Good	0.82	0.41**	0.31***			
Very Good	0.58+	0.33**	0.21***			
Excellent	0.82	0.21**	0.20***			
d) Cognitive Function	0.81*	0.76**	0.66***	0.006	0.008	0.013
e) ADL limitations	1.30***	1.13*	1.29***	0.010	0.004	0.025
f) CRP	1.24***	1.52**	1.08***	0.007	**0.017**	0.006
CRP x Age	--	0.99*	--			

^+^ *p* < 0.10,

* *p* < 0.05,

** *p* < 0.01,

*** *p* < 0.001,

two-tailed.

Note: The best predictor of mortality for a given model in the specified country is indicated with bold type.

^a^ With the exception of self-assessed health status and education, the HR represents the effect per SD of the specified predictor.

^b^ Change in the AUC is based on a comparison between a model that includes the specified predictor with one that excludes that predictor.

^c^ The effect of the predictor varied with age; the main effect represents the HR at age 54.

^d^ In addition to age and sex, sociodemographic control variables include race/ethnicity (except in Costa Rica were race/ethnicity was not asked because 90% of the population is white/mestizo), marital status, and educational attainment.

In order to account for sampling design, the models for Taiwan also included urban residence and the models for Costa Rica included residence in the Nicoya region.

The next set of models controlled for age and again included each of the remaining predictors individually. LTL fell to 17th place in Costa Rica, 21st in Taiwan, and 16th in the U.S. (out of 21). Net of age, the incremental contribution of LTL was substantially smaller (ΔAUC<0.007) than the contributions of the best predictor in each country: self-reported mobility in Costa Rica (ΔAUC = 0.017) and self-assessed health status in Taiwan (ΔAUC = 0.02) and U.S. (ΔAUC = 0.042; Table 3, Model 2). Self-reported mobility was among the top five predictors of mortality in all three countries.

**Fig 2:**
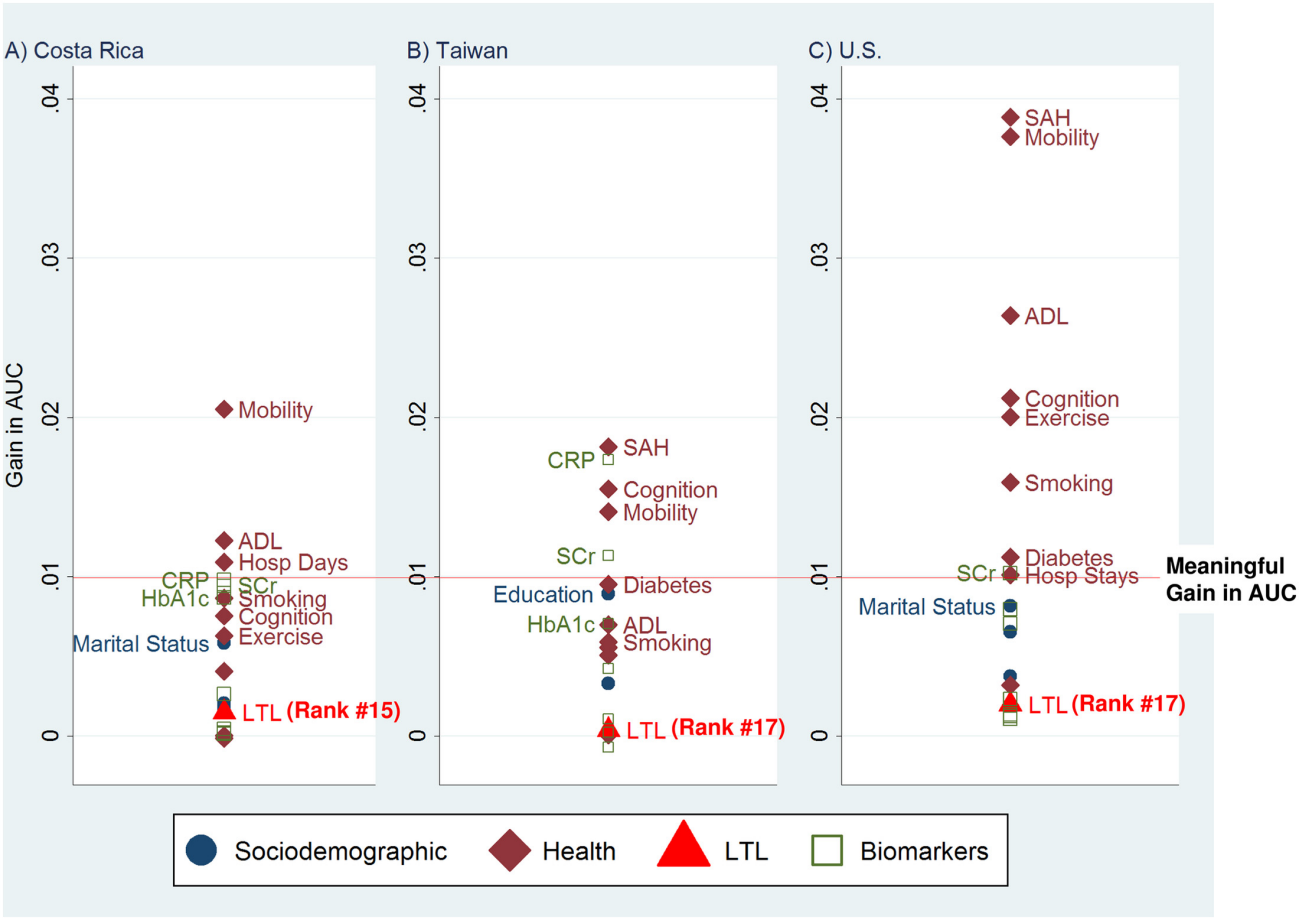
Gain in AUC for 20 Potential Predictors of Five-Year Mortality After Adjustment for Age and Sex. (A) Costa Rica. (B) Taiwan. (C) U.S. Only the top 10 predictors and LTL are labeled. Abbreviations: ADL, Activities of daily living; AUC, Area under the receiver-operating-characteristic curve; CRP, C-reactive protein; HbA1c, Glycosylated hemoglobin; SAH, Self-assessed health status; SCr, Serum creatinine.

Because LTL is strongly correlated with both age and sex [21, 22], we ran a final set of models that adjusted for both age and sex. LTL still ranked low (15th in Costa Rica, 17th in Taiwan and the U.S.; Fig 2) and its incremental contribution was very small (ΔAUC<0.002; Table 4). Net of age and sex, 13 variables were more powerful predictors of mortality than LTL in all three countries: 10 self-reported (mobility, self-assessed health status, ADL limitations, cognitive function, smoking, exercise, hospital stays/days, marital status, education, and social integration) and 3 biomarkers (C-reactive protein, serum creatinine, and glycosylated hemoglobin). Self-reported mobility was consistently one of the best prognostic measures, ranking 1st in Costa Rica (ΔAUC = 0.021), 2nd in the United States (ΔAUC = 0.038), and 4th in Taiwan (ΔAUC = 0.014).

**Table 4:** Gain in AUC after adjustment for age and sex for 20 potential predictors of all-cause mortality, by country.

	Costa Rica (*N =* 923)		Taiwan (*N =* 976)		U.S. (*N =* 2672)	
Predictor	Gain in AUC	Rank	Gain in AUC	Rank	Gain in AUC	Rank
Marital status	0.006	10	0.006	11	0.008	10
Education	0.002	13	0.009	7	0.007	13
Social integration	0.002	14	0.003	15	0.004	14
Smoking status	0.009	7	0.006	10	0.016	6
Exercise frequency	0.006	9	0.005	13	0.020	5
Self-assessed health status	0.004	11	0.018	1	0.039	1
Number of ADL limitations	0.012	2	0.007	9	0.026	3
Index of mobility limitations	0.021	1	0.014	4	0.038	2
History of diabetes	0.000	19	0.010	6	0.011	7
History of cancer	0.000	20	0.000	19	0.003	15
Number of hospital days/stays	0.011	3	0.006	12	0.010	9
Cognitive function	0.008	8	0.015	3	0.021	4
Systolic blood pressure	0.000	16	0.000	18	0.001	18
Diastolic blood pressure	0.003	12	0.001	16	0.001	20
Total cholesterol	0.000	18	0.004	14	0.001	19
Glycosylated hemoglobin	0.009	6	0.007	8	0.008	11
Body mass index	0.000	17	-0.001	20	0.002	16
C-reactive protein	0.010	4	0.017	2	0.007	12
Serum creatinine	0.009	5	0.011	5	0.010	8
Leukocyte telomere length	0.001	15	0.000	17	0.002	17

### Robustness to Alternative Specifications

Because the onset of cellular senescence is triggered by the shortest telomeres [53], LTL may have a non-linear association with mortality. When we categorized LTL and the other biomarkers into quintiles, the results remained similar (see S1, S2 and S3 Figs, Panel B). Net of age and sex, LTL still yielded a small improvement in discrimination (ΔAUC<0.005) in all countries. We found no evidence of a non-linear relationship between average LTL and mortality.

Some have suggested that the association between LTL and mortality may be stronger at younger ages [21, 54]. We explored this hypothesis using all respondents 20 and older for whom LTL was assayed in the U.S. (*N =* 7822; data from Costa Rica and Taiwan did not include younger individuals). We found no evidence that the effect of LTL on mortality varied significantly by age, whether we treated age as linear or categorical: 20–59, 60–74, 75–84, 85+ (see S2 Appendix and S3 Table). The findings based on respondents 20 and older (S4 Fig, Panel A) were similar to those presented here. This consistency results largely from the small number of deaths between ages 20 and 59, many of which result from external causes.

The strength of LTL as a mortality predictor may also vary by length of follow-up. For example, among people who are critically ill, LTL might be inflated because of a shift in the distribution of leukocyte subtypes (e.g., an increase in the proportion of neutrophils, which tend to have longer telomere length than lymphocytes [55]). An increase in LTL just prior to death would weaken the relationship with short-term mortality. It is also plausible that LTL is a stronger predictor of long-term than short-term mortality because telomere length reflects the gradual process of cellular aging. However, our tests for non-proportional hazards showed no evidence that the effect of LTL varied by duration of follow-up in any country. When we excluded deaths within one year after LTL measurement (*N =* 64 in CRELES, *N =* 15 in SEBAS, and *N =* 55 in NHANES) and modeled the association between LTL and mortality from one to five years post-exam, LTL ranked lower relative to the other predictors. After extending the follow-up period to include all available data (mean 6.6 years for CRELES, 11.2 years for SEBAS, and 9.9 years for NHANES) and including all U.S. respondents aged 20 and older, we found that LTL contributed a negligible improvement in the AUC (<0.001) net of age and sex. In sum, we found little evidence that the association between LTL and mortality differed by length of follow-up.

Next, we re-estimated the models adjusting for a broader set of sociodemographic control variables, including race/ethnicity, marital status, and education. The results were essentially unchanged: LTL still ranked low and the incremental improvement in the AUC net of age and sex remained very small (Table 3, Model 4 and S1, S2 and S3 Figs, Panel C).

Finally, results from a similar set of models using cause-specific mortality (i.e., cardiovascular disease, cancer, and all other causes combined; see S2 Appendix and S4 Table) as the outcome were consistent with those presented here for all-cause mortality. Net of age and sex, LTL yielded a negligible improvement in the AUC and ranked well below many other predictors (S5 Table and S4, S5 and S6 Figs).

## Discussion

Technological advances allow us to measure intricate details about human physiology. Yet, to prove its worth, a novel biomarker should tell us more than we already know based on simpler observables. The biological processes of telomere shortening and subsequent cell senescence suggest potentially strong linkages between telomere length, aging, and survival. Previous efforts that have identified statistically significant linkages between telomere length and mortality seemingly provide some support for this expectation. However, prior to our analysis, the discriminatory ability of telomere length relative to well-established variables in the social and health sciences had never been evaluated.

Consistent with several studies that examined the relationship between LTL and five-year all-cause mortality, we found a significant hazard ratio in an unadjusted model, although the effect size was modest and was largely attenuated after controlling for age (Table 3). More importantly for the purpose of prognosis, we found that LTL had little discriminatory ability and under-performed many conventional predictors of mortality, including easily collected self-reported measures. Indeed, 13 variables were more powerful predictors of mortality than LTL in all three countries. The self-reported measure of mobility limitations was consistently one of the strongest predictors of all-cause mortality. Given that strong mortality predictors are almost certainly proxies for myriad factors accumulated over a lifetime, the subjective nature of self-reports has the advantage of capturing perceptions that may integrate complex information.

The weak contribution of LTL may result, at least in part, from the difficulty of measurement. There is notable measurement error in LTL analysis resulting from various sources including DNA quality and within- and between-well and -plate error [56]. In addition, normal day-to-day variation in LTL represents noise that reduces statistical power to isolate the underlying signal [57]. Random variation—whether it results from measurement error or other factors—will lead to attenuation bias. If non-systematic error is greater for LTL than for other variables, it would reduce the relative ranking of LTL. Importantly, the inter-assay coefficient of variation of the three studies reported here ranges from 4 to 7%, which is on the lower end of the reported variation for Q-PCR telomere length assays [56].

A further limitation is that this study evaluates only a one-time measurement of LTL. We do not have the data to quantify individual-level changes in LTL over time for all three datasets, and thus cannot assess whether the rate of telomere shortening might provide more prognostic power. One important challenge in estimating the effects of LTL attrition is that measurement error becomes a much bigger problem. If there is substantial measurement error, the apparent change in LTL may reflect more noise than signal, and statistical power would be severely compromised.

An additional concern is that our analysis is limited to telomere length in leukocytes, which may not reflect telomere length in other tissues. Although telomere lengths from various tissues are well-correlated [1], that may not hold across all tissue types. Furthermore, the distribution of leukocyte (white blood cell, WBC) subtypes may affect measures of LTL because the Q-PCR technique yields a weighted average across a mix of different cell types. Although highly correlated, different WBC subsets have different telomere lengths [55, 58, 59]. However, Glei et al. [7] showed that controlling for WBC distribution had little effect on the association between LTL and mortality.

Evidence suggests that it is the shortest telomeres, rather than average telomere length, that determine the onset of cellular senescence [53]. Using the Terminal Restriction Fragment method, Kimura et al. [37] found that the average length of the shortest telomeres was a better predictor of mortality than the overall average LTL. Unfortunately, the Q-PCR technique used to assay LTL in our study does not provide information about the distribution of telomere lengths.

The finding that LTL is a weak predictor of mortality in the general population might be explained in part by competing risks. Both extrinsic risk factors and other intrinsic processes may lead to death long before they lead to substantial telomere shortening. The association between LTL and mortality may appear stronger in healthy subpopulations where many competing risks are dormant. However, in exploratory analyses that excluded respondents who reported poor or fair health, the increase in AUC attributable to LTL continued to be very small (results not shown).

Finally, we have evaluated LTL and the other variables *only* in terms of their ability to predict mortality. This constraint entails two important limitations. First, while LTL is not a strong predictor of mortality among older people, it could be a valuable marker of healthspan or of particular aging-related diseases. LTL has been associated with multiple diseases of aging, with the strongest associations for coronary heart disease [60]. One study reported that short LTL was associated with fewer years of healthy life, but not with shorter lifespan, supporting the notion that LTL might be a biomarker of healthy aging, but not a biomarker of survival [31]. However, our auxiliary analyses of cause-specific mortality (i.e., cardiovascular, cancer, and all other causes combined) suggest that LTL does not perform well against other predictors. Second, the best predictors do not necessarily have causal effects on mortality; they may not represent root causes that can be modified or treated to lower the risk of premature mortality. Nevertheless, accurate prognosis is important when patients and their doctors weigh the risks and benefits of a given treatment.

We find that the molecular clock is nowhere near as powerful as chronological age when it comes to predicting five-year mortality—at least among older humans. Net of age and sex, numerous variables were better predictors of mortality than LTL including self-reported mobility, self-assessed health status, an assessment of cognitive function, smoking, exercise, an inflammatory marker (C-reactive protein), and a marker of kidney function (serum creatinine). Although LTL may eventually help scientists understand aging, more powerful and more easily obtained tools are available for predicting survival.

## Supporting Information

S1 Appendix Additional Details Regarding Methods. (DOCX)

S2 Appendix Supplementary Results. (DOCX)

S1 Data Stata (version 12.1) dataset with CRELES variables used in this analysis (*N* = 923). (DTA)

S1 Fig Predictors of Five-Year All-Cause Mortality After Adjustment for Age and Sex Ranked by the Gain in AUC, Comparison with Alternative Specifications, Costa Rica (*N* = 934, Aged 61+). A, Same model shown in Fig 2(A). B, Biomarkers specified as categorical (quintiles). C, Adjusted for additional sociodemographic variables (i.e., marital status, education, and Nicoya region). Only the top 10 predictors and LTL are labeled. Abbreviations: ADL, Activities of daily living; AUC, Area under the receiver-operating-characteristic curve; CRP, C-reactive protein; HbA1c, Glycosylated hemoglobin; LTL, Leukocyte telomere length; SAH, Self-assessed health status; SBP, Systolic blood pressure; SCr, Serum creatinine.
(DOCX)

S2 Fig Predictors of Five-Year All-Cause Mortality After Adjustment for Age and Sex Ranked by the Gain in AUC, Comparison with Alternative Specifications, Taiwan (*N* = 976, Aged 54+). A, Same model shown in Fig 2(B). B, Biomarkers specified as categorical (quintiles). C, Adjusted for additional sociodemographic variables (i.e., ethnicity, marital status, education, and urban). Only the top 10 predictors and LTL are labeled. Abbreviations: ADL, Activities of daily living; AUC, Area under the receiver-operating-characteristic curve; CRP, C-reactive protein; HbA1c, Glycosylated hemoglobin; LTL, Leukocyte telomere length; SAH, Self-assessed health status; SCr, Serum creatinine, TC, Total cholesterol.
(DOCX)

S3 Fig Predictors of Five-Year All-Cause Mortality After Adjustment for Age and Sex Ranked by the Gain in AUC, Comparison with Alternative Specifications, U.S. (*N* = 3672, Aged 60+). A, Same model shown in Fig 2(C). B, Biomarkers specified as categorical (quintiles). C, Adjusted for additional sociodemographic variables (i.e., race/ethnicity, marital status, and education). Only the top 10 predictors and LTL are labeled. Abbreviations: ADL, Activities of daily living; AUC, Area under the receiver-operating-characteristic curve; CRP, C-reactive protein; HbA1c, Glycosylated hemoglobin; LTL, Leukocyte telomere length; SAH, Self-assessed health status; SCr, Serum creatinine.
(DOCX)

S4 Fig Predictors of Cause-Specific Mortality After Adjustment for Age and Sex, Ranked by the Gain in AUC, U.S. (*N* = 7822, Aged 20+). A, All causes. B, Cardiovascular. C, Cancer. D, All other causes. Only the top 10 predictors and LTL are labeled. Abbreviations: ADL, Activities of daily living; AUC, Area under the receiver-operating-characteristic curve; HbA1c, Glycosylated hemoglobin; LTL, Leukocyte telomere length; SAH, Self-assessed health status; SBP, Systolic blood pressure; SCr, Serum creatinine.
(DOCX)

S5 Fig Predictors of Cause-Specific Mortality After Adjustment for Age and Sex, Ranked by the Gain in AUC, Costa Rica (*N* = 923, Aged 61+). A, All causes. B, Cardiovascular. C, Cancer. D, All other causes. Only the top 10 predictors and LTL are labeled. Abbreviations: ADL, Activities of daily living; AUC, Area under the receiver-operating-characteristic curve; CRP, C-reactive protein; DBP, Diastolic blood pressure; HbA1c, Glycosylated hemoglobin; LTL, Leukocyte telomere length; SAH, Self-assessed health status; SBP, Systolic blood pressure; SCr, Serum creatinine.
(DOCX)

S6 Fig Predictors of Cause-Specific Mortality After Adjustment for Age and Sex, Ranked by the Gain in AUC, Taiwan (*N* = 976, Aged 54+). A, All causes. B, Cardiovascular. C, Cancer. D, All other causes. Only the top 10 predictors and LTL are labeled. Abbreviations: ADL, Activities of daily living; AUC, Area under the receiver-operating-characteristic curve; CRP, C-reactive protein; DBP, Diastolic blood pressure; HbA1c, Glycosylated hemoglobin; LTL, Leukocyte telomere length; TC, Total cholesterol; SAH, Self-assessed health status; SBP, Systolic blood pressure; SCr, Serum creatinine.
(DOCX)

S1 Table Variables Included in Index of Social Integration for Each Dataset. (DOCX)

S2 Table Descriptive Statistics for All Analysis Variables, Weighted. (DOCX)

S3 Table Models Testing for Age-Dependent Effects of LTL on All-Cause Mortality. NHANES (U.S.), Ages 20 and Older (*N* = 7,822).
(DOCX)

S4 Table Analysis Samples for Cause-Specific Mortality Using All Available Data. (DOCX)

S5 Table Hazard Ratios (HR) and Gain in AUC Attributable to LTL and the Best Predictors^a^ of Cause-Specific Mortality Adjusted for Age and Sex. CRELES (Costa Rica, ***N* =** 923, aged 61+), SEBAS (Taiwan, ***N* =** 976, aged 54+), and NHANES (U.S., ***N* =** 7822, aged 20+).
(DOCX)
