# Supplementary material for: Predicting Survival from Telomere Length versus Conventional Predictors: A Multinational Population-Based Cohort Study
Source: PLoS One. 2016 Apr 6;11(4):e0152486. doi: 10.1371/journal.pone.0152486 (PMC4822878; doi:10.1371/journal.pone.0152486)
Supplement: S1 Appendix — (DOCX) [file pone.0152486.s001.docx]

# S1 Appendix. Additional Details Regarding Methods.

## Mortality Follow-up

For NHANES, vital status as of January 1, 2012 was determined by linkage with death certificate records from the National Death Index. For SEBAS, survival status as of January 1, 2012 was ascertained through linkage with the death certificate file maintained by the Department of Health and the household registration database maintained by the Ministry of the Interior. For CRELES, survival status as of March 31, 2014 was established in two ways: (1) through the computer records in the National Death Registry, and (2) during the third (2008-2010) wave of home visits. The computer follow-up used the unique identification number (the *cédula*) assigned to Costa Ricans. For the foreigners in the sample (approx. 3%), survival was established only in the field because they did not have a unique identification number with which to link them to the Registry.

## Leukocyte Telomere Length (LTL) Assay Protocols

In CRELES, laboratories at the University of Costa Rica extracted DNA from 2 ml of frozen whole blood using the phenol-chloroform method. LTL was assayed by the Blackburn laboratory at the University of California, San Francisco using quantitative polymerase chain reaction (Q-PCR) to determine the relative ratio of telomere to a single-copy gene (T/S ratio), in this case human beta-globin. Mean LTL was based on an average of two assays per DNA extraction sample. The laboratories were blinded to the sample characteristics. The inter-assay coefficient of variability (CV) for LTL was 3.7%. LTL assays of the stored DNA were conducted in two batches, with the first batch run in 2010 and the second in 2014. To make the results from the two batches more comparable, a linear adjustment was applied based on a validation lot of 30 DNA samples included in both batches. The correlation coefficient between the two measures of LTL for the validation lot was 0.94. We tested for possible batch effects and found no evidence that the effect of LTL on mortality varied by batch.

For NHANES, DNA was extracted from whole blood using standardized methods. The LTL assay was performed in the Blackburn laboratory at the University of California, San Francisco, using Q-PCR to measure the T/S ratio, as described elsewhere [58, 59]. Human beta-globin was the single-copy gene used to normalize input DNA [60]. Each sample was assayed three times on three different days. After excluding potential outliers (< 2% of samples), the mean LTL was calculated as the average of the remaining values. The inter-assay CV was 6.5%.

In SEBAS, DNA was extracted by Union Clinical Laboratories (in Taiwan) using trimethyl ammonium bromide salts (DTAB and CTAB). LTL, represented by the T/S ratio, was measured at the University of Washington, using Q-PCR with a few modifications from the method originally developed by Cawthon [58, 61]. For each sample, two PCRs were performed: the first one to amplify the telomeric DNA and the second one to amplify a single-copy control gene (36B4, acidic ribosomal phosphoprotein PO), which provided an internal control to normalize the starting amount of DNA. All samples were run in triplicate and the median was used for subsequent calculations. The amount of telomeric DNA (T) was divided by the amount of control gene DNA (S), producing a relative measurement of the telomere length of the sample (T/S). Two control samples were run in each experiment to allow for normalization between experiments and periodic reproducibility experiments were performed to guarantee correct measurements. The inter-assay CV was 7%.

## Multiple Imputation Procedures

Data were missing for at least one of the predictors among 33% of the CRELES sample, 15% of the NHANES sample, and 6% of the SEBAS sample. The individual variables that were missing for more than 2% of the sample included: marital status (in NHANES), mobility limitations (in CRELES),^[[1]](#footnote-1)^ cognitive function (in CRELES), blood pressure (in NHANES), and body mass index (in CRELES and NHANES). Following standard practices of multiple imputation [62, 63], we created five imputed datasets using regression techniques to fill in missing values; the predictors comprised all of the variables in this analysis plus several auxiliary variables that were correlated with non-response (e.g., interviewed by proxy, high-density lipoprotein cholesterol, waist circumference, race/ethnicity). Then, we estimated the model for each imputed dataset and combined the five sets of estimates using Rubin’s rules [64]. All measures of fit and predictive ability were calculated for each dataset and then averaged following the same rules.

1. Nearly 13% of the CRELES sample were missing mobility limitations because a substantial proportion of respondents reported that they “do not do it” when asked about difficulty performing some tasks (e.g., climbing stairs, pushing/pulling large objects). Such responses were coded as missing because we do not know whether the respondent is actually “unable” to do that task or simply avoids it. In the multiple imputation prediction equations we included each of the individual tasks as predictors (because the majority is missing only one of the four mobility task items; only one respondent is missing all four). [↑](#footnote-ref-1)
