# Supplementary material for: Predicting Survival from Telomere Length versus Conventional Predictors: A Multinational Population-Based Cohort Study
Source: PLoS One. 2016 Apr 6;11(4):e0152486. doi: 10.1371/journal.pone.0152486 (PMC4822878; doi:10.1371/journal.pone.0152486)
Supplement: S2 Appendix — (DOCX) [file pone.0152486.s002.docx]

# S2 Appendix. Supplementary Results

## Does LTL have an age-dependent effect?

To investigate whether the effect of LTL varied by age, we first tested for an interaction between age (treated as linear) and LTL. Neither the main effect for LTL nor the interaction between LTL and age was significant (see S3 Table, Model 1). Secondly, to allow for the possibility that the effect of LTL may vary with age in a non-linear pattern, we split the exposure for each respondent into the following age groups: 20-59, 60-74, 75-84, 85+. Then, we tested whether the effect of LTL differed significantly by age group. The joint χ^2^ test of the interaction terms was not significant (*p*~0.15), and the pattern of the coefficients did not follow a consistent pattern (S3 Table, Model 2).

## Predicting Cause-Specific Mortality

While the focus of this paper is on all-cause mortality, we have conducted similar models for three groups of causes: cardiovascular disease (CVD), malignant neoplasms, and all other causes. In order to maximize statistical power, we used the full sample of respondents aged 20 and older from NHANES and all available follow-up from each country (S4 Table). Despite grouping causes in broad categories and using all available data, we have a relatively small number of deaths from CVD and cancer in Costa Rica and Taiwan. NHANES provides, by far, the most statistical power for modeling cause-specific mortality.

Hazard ratios (HRs) and gains in AUC attributable to LTL and other selected best predictors are shown in S5 Table for mortality from each cause group. Each predictor was tested individually controlling only for age (as the clock) and sex. The HR was significant (albeit small in magnitude) only for all-cause mortality in the NHANES sample. Nonetheless, even in this case, LTL produced a negligible improvement in predictive ability (ΔAUC<0.001) net of age and sex. Relative to the other predictors (Figures S4-S6), LTL ranked near the bottom in predicting all outcomes considered here (all-cause, CVD, cancer or other mortality).
