# Supplementary material for: Predicting Survival from Telomere Length versus Conventional Predictors: A Multinational Population-Based Cohort Study
Source: PLoS One. 2016 Apr 6;11(4):e0152486. doi: 10.1371/journal.pone.0152486 (PMC4822878; doi:10.1371/journal.pone.0152486)
Supplement: S1 Fig — A, Same model shown in Fig 2(A). B, Biomarkers specified as categorical (quintiles). C, Adjusted for additional sociodemographic variables (i.e., marital status, education, and Nicoya region). Only the top 10 predictors and LTL are labeled. Abbreviations: ADL, Activities of daily living; AUC, Area under the receiver-operating-characteristic curve; CRP, C-reactive protein; HbA1c, Glycosylated hemoglobin; LTL, Leukocyte telomere length; SAH, Self-assessed health status; SBP, Systolic blood pressure; SCr, Serum creatinine. (DOCX) [file pone.0152486.s004.docx]

**S1 Fig.**  **Predictors of Five-Year All-Cause Mortality After Adjustment for Age and Sex Ranked by the Gain in AUC, Comparison with Alternative Specifications, Costa Rica (*N*=934, Aged 61+).** A) Same model shown in Figure 2(A). (B) Biomarkers specified as categorical (quintiles). (C) Adjusted for additional sociodemographic variables (i.e., marital status, education, and Nicoya region). Only the top 10 predictors and LTL are labeled.

Abbreviations: ADL, Activities of daily living; AUC, area under the receiver-operating-characteristic curve; CRP, C-reactive protein; HbA1c, Glycosylated hemoglobin; LTL, Leukocyte telomere length; SAH, Self-assessed health status; SBP, Systolic blood pressure; SCr, Serum creatinine.

Meaningful

Gain in AUC
