# Supplementary material for: Predicting Survival from Telomere Length versus Conventional Predictors: A Multinational Population-Based Cohort Study
Source: PLoS One. 2016 Apr 6;11(4):e0152486. doi: 10.1371/journal.pone.0152486 (PMC4822878; doi:10.1371/journal.pone.0152486)
Supplement: S2 Fig — A, Same model shown in Fig 2(B). B, Biomarkers specified as categorical (quintiles). C, Adjusted for additional sociodemographic variables (i.e., ethnicity, marital status, education, and urban). Only the top 10 predictors and LTL are labeled. Abbreviations: ADL, Activities of daily living; AUC, Area under the receiver-operating-characteristic curve; CRP, C-reactive protein; HbA1c, Glycosylated hemoglobin; LTL, Leukocyte telomere length; SAH, Self-assessed health status; SCr, Serum creatinine, TC, Total cholesterol. (DOCX) [file pone.0152486.s005.docx]

**S2 Fig.**  **Predictors of Five-Year All-Cause Mortality After Adjustment for Age and Sex Ranked by the Gain in AUC, Comparison with Alternative Specifications, Taiwan (*N*=976, Aged 54+).** (A) Same model shown in Figure 2(B). (B) Biomarkers specified as categorical (quintiles). (C) Adjusted for additional sociodemographic variables (i.e., ethnicity, marital status, education, and urban). Only the top 10 predictors and LTL are labeled.

Abbreviations: ADL, Activities of daily living; AUC, area under the receiver-operating-characteristic curve; CRP, C-reactive protein; HbA1c, Glycosylated hemoglobin; LTL, Leukocyte telomere length; SAH, Self-assessed health status; SCr, Serum creatinine, TC, Total cholesterol.

Meaningful

Gain in AUC
