# Supplementary material for: Predicting Survival from Telomere Length versus Conventional Predictors: A Multinational Population-Based Cohort Study
Source: PLoS One. 2016 Apr 6;11(4):e0152486. doi: 10.1371/journal.pone.0152486 (PMC4822878; doi:10.1371/journal.pone.0152486)
Supplement: S3 Fig — A, Same model shown in Fig 2(C). B, Biomarkers specified as categorical (quintiles). C, Adjusted for additional sociodemographic variables (i.e., race/ethnicity, marital status, and education). Only the top 10 predictors and LTL are labeled. Abbreviations: ADL, Activities of daily living; AUC, Area under the receiver-operating-characteristic curve; CRP, C-reactive protein; HbA1c, Glycosylated hemoglobin; LTL, Leukocyte telomere length; SAH, Self-assessed health status; SCr, Serum creatinine. (DOCX) [file pone.0152486.s006.docx]

**S3 Fig.**  **Predictors of Five-Year All-Cause Mortality After Adjustment for Age and Sex Ranked by the Gain in AUC, Comparison with Alternative Specifications, U.S. (*N*=3672, Aged 60+).** (A) Same model shown in Figure 2(C). (B) Biomarkers specified as categorical (quintiles). (C) Adjusted for additional sociodemographic variables (i.e., race/ethnicity, marital status, and education). Only the top 10 predictors and LTL are labeled.

Abbreviations: ADL, Activities of daily living; AUC, area under the receiver-operating-characteristic curve; CRP, C-reactive protein; HbA1c, Glycosylated hemoglobin; LTL, Leukocyte telomere length; SAH, Self-assessed health status; SCr, Serum creatinine.

Meaningful

Gain in AUC
