# Supplementary material for: Predicting Survival from Telomere Length versus Conventional Predictors: A Multinational Population-Based Cohort Study
Source: PLoS One. 2016 Apr 6;11(4):e0152486. doi: 10.1371/journal.pone.0152486 (PMC4822878; doi:10.1371/journal.pone.0152486)
Supplement: S5 Fig — A, All causes. B, Cardiovascular. C, Cancer. D, All other causes. Only the top 10 predictors and LTL are labeled. Abbreviations: ADL, Activities of daily living; AUC, Area under the receiver-operating-characteristic curve; CRP, C-reactive protein; DBP, Diastolic blood pressure; HbA1c, Glycosylated hemoglobin; LTL, Leukocyte telomere length; SAH, Self-assessed health status; SBP, Systolic blood pressure; SCr, Serum creatinine. (DOCX) [file pone.0152486.s008.docx]

**S5 Fig.**  **Predictors of Cause-Specific Mortality After Adjustment for Age and Sex, Ranked by the Gain in AUC, Costa Rica (*N*=923, Aged 61+). (**A) All causes. (B) Cardiovascular. (C) Cancer. (D) All other causes. Only the top 10 predictors and LTL are labeled. Abbreviations: ADL, Activities of daily living; AUC, area under the receiver-operating-characteristic curve; CRP, C-reactive protein; DBP, Diastolic blood pressure; HbA1c, Glycosylated hemoglobin; LTL, Leukocyte telomere length; SAH, Self-assessed health status; SBP, Systolic blood pressure; SCr, Serum creatinine.

Meaningful

Gain in AUC
