# Supplementary material for: Predicting Survival from Telomere Length versus Conventional Predictors: A Multinational Population-Based Cohort Study
Source: PLoS One. 2016 Apr 6;11(4):e0152486. doi: 10.1371/journal.pone.0152486 (PMC4822878; doi:10.1371/journal.pone.0152486)
Supplement: S1 Table — (DOCX) [file pone.0152486.s010.docx]

**S1 Table. Variables Included in Index of Social Integration for Each Dataset.**

| **Variable** | **Definition and Coding** | **CRELES**  **Wave 2** | **NHANES**  **1999-2002** | **SEBAS 2000** |
| --- | --- | --- | --- | --- |
| Network size | Number of friends/relatives the respondent lives with or has regular contact; recoded <5, 5-7, 8-10, 11-14, 15-19, 20-29, 30+ | N/A | N/A | ✓ |
| Network range | Number of types of relationships in social network; One point each for spouse/partner, kids, other relatives, non-relatives; range: 0-4 | N/A | N/A | ✓ |
| Frequency of interaction  with network members | How often the respondent has contact with friends, neighbors, or relatives; Response categories from 0=never to 4=nearly daily | N/A | N/A | ✓ |
| Household size | Top-coded at 7+ | ✓ | ✓ | ✓ |
| Does not live alone | Dummy indicating that the respondent does not live alone | ✓ | ✓ | ✓ |
| Married/partner | Dummy indicating that the respondent is married or lives with a companion | ✓ | ✓ | ✓ |
| Number of friends | NHANES: Number of close friends; recoded 0, 1-2, 3-4, 5-9, 10-19, 20+  SEBAS: Number of close friends and neighbors with whom the respondent has weekly contact; recoded 0, 1-2, 3-4, 5-9, 10-19, 20+ | N/A | ^a^ | ✓ |
| Number of children | Top-coded at 8+ | ✓ | N/A | ✓ |
| Number of grandchildren | CRELES: recoded 0, 1-4, 5-9, 10-14,15-24, 25+  SEBAS: recoded 0, 1-2, 3-5, 6-9, 10-14, 15+ | ✓ | N/A | ✓ |
| Religious attendance | How often the respondent attends religious services; Response categories from 0=never to 3=often (SEBAS); to 4=more than once a week (CRELES) | ^a^ | N/A | ✓ |
| Participation in  social organizations | CRELES: Summed number of hours per week spent in church and civic activities and then categorized into 0, 1, 2, 3-4, 5+ hours per week  SEBAS: One point for each of 8 social organizations/activities in which the respondent participates | ^a^ | N/A | ✓ |

^a^ Item was dropped from the index because the item-rest correlation was less than 0.20.
