# Supplementary material for: Predicting Survival from Telomere Length versus Conventional Predictors: A Multinational Population-Based Cohort Study
Source: PLoS One. 2016 Apr 6;11(4):e0152486. doi: 10.1371/journal.pone.0152486 (PMC4822878; doi:10.1371/journal.pone.0152486)
Supplement: S2 Table — (DOCX) [file pone.0152486.s011.docx]

**S2 Table. Descriptive Statistics for All Analysis Variables, Weighted.**

|  | **Costa Rica [CRELES]**  **(*N=*923)** | | |  | **Taiwan [SEBAS]**  **(*N=*976)** | | |  | **U.S. [NHANES]**  **(*N=*2672)** | | |
| --- | --- | --- | --- | --- | --- | --- | --- | --- | --- | --- | --- |
|  | Mean  or % | SD | Range |  | Mean  or % | SD | Range |  | Mean  or % | SD | Range |
| Died within five years post-exam, % | 19.8 |  |  |  | 11.4 |  |  |  | 14.4 |  |  |
| **Demographic** |  |  |  |  |  |  |  |  |  |  |  |
| Age at exam | 72.4 | 8.1 | 61-111 |  | 67.0 | 8.1 | 54-92 |  | 71.6 | 8.0 | 60-90^a^ |
| Female, % | 54.6 |  |  |  | 43.8 |  |  |  | 55.5 |  |  |
| **Social factors** |  |  |  |  |  |  |  |  |  |  |  |
| Marital status |  |  |  |  |  |  |  |  |  |  |  |
| Married, % | 57.1 |  |  |  | 74.9 |  |  |  | 61.8 |  |  |
| Widowed, % | 23.0 |  |  |  | 20.6 |  |  |  | 25.6 |  |  |
| Divorced/separated, % | 13.9 |  |  |  | 1.7 |  |  |  | 9.7 |  |  |
| Never married, % | 6.0 |  |  |  | 2.9 |  |  |  | 2.9 |  |  |
| Education^b^ |  |  |  |  |  |  |  |  |  |  |  |
| Very low | 15.3 |  |  |  | 32.6 |  |  |  | 14.4 |  |  |
| Low | 14.8 |  |  |  | 8.8 |  |  |  | 16.6 |  |  |
| Medium | 26.2 |  |  |  | 34.4 |  |  |  | 28.9 |  |  |
| High | 28.3 |  |  |  | 9.5 |  |  |  | 21.2 |  |  |
| Very high | 15.5 |  |  |  | 14.7 |  |  |  | 19.0 |  |  |
| Index of social integration | -0.02 | 0.61 | -1.7 to 1.2 |  | 0.1 | 0.5 | -1.7 to 1.5 |  | -0.4 | 0.9 | -2.1 to 1.1 |
| Health behaviors |  |  |  |  |  |  |  |  |  |  |  |
| Smoking status |  |  |  |  |  |  |  |  |  |  |  |
| Never, % | 56.2 |  |  |  | 59.2 |  |  |  | 47.0 |  |  |
| Former, % | 35.6 |  |  |  | 17.1 |  |  |  | 40.8 |  |  |
| Current, % | 8.2 |  |  |  | 23.7 |  |  |  | 12.2 |  |  |
| (continued on next page) |  |  |  |  |  |  |  |  |  |  |  |
| Exercise frequency |  |  |  |  |  |  |  |  |  |  |  |
| 3+ times per week, % | 27.5 |  |  |  | -- |  |  |  | -- |  |  |
| No exercise, % | -- |  |  |  | 38.5 |  |  |  | 50.0 |  |  |
| < 3 times per week, % | -- |  |  |  | 9.9 |  |  |  | -- |  |  |
| 3-5 times per week, % | -- |  |  |  | 13.3 |  |  |  | -- |  |  |
| 6+ times per week, % | -- |  |  |  | 38.3 |  |  |  | -- |  |  |
| < 12 times in 30 days, % | -- |  |  |  | -- |  |  |  | 14.1 |  |  |
| 12-29 times in 30 days, % | -- |  |  |  | -- |  |  |  | 15.9 |  |  |
| 30+ times in 30 days, % | -- |  |  |  | -- |  |  |  | 19.9 |  |  |
| Health status |  |  |  |  |  |  |  |  |  |  |  |
| Self-assessed health status | 2.8 | 1.0 | 1-5 |  | 3.1 | 1.0 | 1-5 |  | 3.3 | 1.1 | 1-5 |
| Number of ADL limitations | 0.4 | 1.1 | 0-5 |  | 0.1 | 0.6 | 0-6 |  | 0.6 | 1.2 | 0-5 |
| Index of mobility limitations | 0.2 | 0.9 | -0.7 to 2.1 |  | 0.4 | 1.2 | -0.7 to 3.2 |  | 0.7 | 1.3 | -0.7 to 3.2 |
| History of diabetes, % | 26.0 |  |  |  | 15.0 |  |  |  | 14.5 |  |  |
| History of cancer, % | 5.3 |  |  |  | 2.6 |  |  |  | 20.6 |  |  |
| Number of hospital stays | -- |  |  |  | 0.2 | 0.7 | 0-10 |  | 0.2 | 0.6 | 0-10 |
| Number of hospital days | 1.3 | 6.7 | 0-97 |  | -- |  |  |  | -- |  |  |
| Cognitive function | 89.2 | 10.7 | 8-100 |  | 16.5 | 3.8 | 0-24 |  | 42.8 | 21.6 | 0-117 |
| Biomarkers |  |  |  |  |  |  |  |  |  |  |  |
| Systolic blood pressure (mmHg) | 143.4 | 23.1 | 73-240 |  | 137.8 | 20.8 | 83-243 |  | 141.3 | 22.7 | 82-233 |
| Diastolic blood pressure (mmHg) | 81.3 | 12.1 | 45-131 |  | 82.6 | 11.3 | 50-141 |  | 69.5 | 16.0 | 0-122 |
| Total cholesterol (mmol/L) | 5.3 | 1.1 | 1.5-10.7 |  | 5.2 | 1.0 | 2.1-9.6 |  | 5.5 | 1.0 | 1.9-12.4 |
| Glycosylated hemoglobin (proportion) | 0.06 | 0.01 | 0.04-0.14 |  | 0.06 | 0.01 | 0.04-0.13 |  | 0.06 | 0.01 | 0.04-0.19 |
| Body mass index | 26.6 | 5.0 | 13.0-50.5 |  | 24.5 | 3.6 | 16.1-42.2 |  | 28.2 | 5.5 | 15.2-52.0 |
| C-reactive protein (nmol/L) | 56.8 | 86.3 | 1.9-606.7 |  | 29.1 | 65.2 | 0.04-874.6 |  | 51.6 | 90.9 | 1.0-2819.1 |
| Serum creatinine (μmol/L) | 94.5 | 53.1 | 31.8-873.4 |  | 89.5 | 36.8 | 44.2-884 |  | 81.9 | 45.3 | 26.5-1043.1 |
| Leukocyte telomere length (T/S ratio) | 1.0 | 0.2 | 0.3-2.1 |  | 0.9 | 0.2 | 0.1-1.8 |  | 0.9 | 0.2 | 0.4-3.0 |

^a^ In order to protect confidentiality, respondents aged 85 and older were top-coded in the NHANES public-use dataset. We have coded age for these respondents to the sex-specific mean age (88.7 for men, 89.5 for women) among the U.S. population aged 85 and older (on 1/1/2000 for the 1999-2000 wave; on 1/1/2002 for the 2001-02 wave) based on population estimates the Human Mortality Database [65].

^b^ Given that educational attainment varied across the three countries, we specified the categories based on the within-country distribution (see Table 1 for details).
