# Supplementary material for: Predicting Survival from Telomere Length versus Conventional Predictors: A Multinational Population-Based Cohort Study
Source: PLoS One. 2016 Apr 6;11(4):e0152486. doi: 10.1371/journal.pone.0152486 (PMC4822878; doi:10.1371/journal.pone.0152486)
Supplement: S3 Table — NHANES (U.S.), Ages 20 and Older (N = 7,822). (DOCX) [file pone.0152486.s012.docx]

**S3 Table. Models Testing for Age-Dependent Effects of LTL on All-Cause Mortality.** NHANES (U.S.), Ages 20 and Older (*N*=7,822).

|  | **β^a^** | **95% C.I.** | **HR^a^** |
| --- | --- | --- | --- |
| **Model 1:** Interacted with Age (linear) |  |  |  |
| LTL (effect at age 20) | 0.078 | -0.138 to 0.295 | 1.08 |
| LTL x age (in years) | -0.003 | -0.007 to 0.008 | 0.997 |
|  |  |  |  |
| **Model 2:** Interacted with Age (categorical)^b^ |  |  |  |
| LTL at ages 20-59 | 0.038 | -0.106 to 0.182 | 1.04 |
| LTL at ages 60-74 | -0.186 | -0.347 to -0.025 | 0.83*^c^ |
| LTL at ages 75-84 | -0.046 | -0.188 to 0.095 | 0.96 |
| LTL at ages 85+ | -0.142 | -0.278 to -0.006 | 0.87* |
| Joint χ^2^ test of interaction terms | χ2=5.28, df=3, *p*~0.15 |  |  |

* *p* < 0.05, two-tailed.

^a^ Standardized effect size per SD of LTL.

^b^ To ease interpretation, the coefficients have been reparameterized to show the effect of LTL for each age group (e.g., the effect for ages 60-74 is the sum of the main effect and the interaction term for that age group).

^c^ The coefficient for ages 60-74 differed significantly (p<0.05) from the coefficient at ages 20-59. None of the other five differences between pairs of coefficients were significant.

Note: These analyses are based on the sample of 7,822 respondents aged 20 and older, 1205 of whom died by the end of the follow-up period (mean 9.8 years, range 8.9-10.9 years). There were 157 deaths at ages 20-59, 277 deaths at ages 60-74, 351 deaths at ages 75-84, and 420 deaths at ages 85 and older. In addition to age and LTL, models control for sex.
