# Supplementary material for: Predicting Survival from Telomere Length versus Conventional Predictors: A Multinational Population-Based Cohort Study
Source: PLoS One. 2016 Apr 6;11(4):e0152486. doi: 10.1371/journal.pone.0152486 (PMC4822878; doi:10.1371/journal.pone.0152486)
Supplement: S4 Table — (DOCX) [file pone.0152486.s013.docx]

S4 Table. Analysis Samples for Cause-Specific Mortality Using All Available Data.

|  | **Costa Rica**  **(*N*=923)** | **Taiwan**  **(*N*=976)** | **U.S.**  **(*N*=7822)** |
| --- | --- | --- | --- |
| Age range of sample | 61+ | 54+ | 20+ |
| Length of mortality follow-up (years),  mean (range) | 6.6 (0.1-7.5 ) | 11.2 (11.1-11.4) | 9.8 (8.9-10.9) |
| Number of deaths from: |  |  |  |
| All causes | 336 | 340 | 1205 |
| Cardiovascular disease (CVD)^a^ | 105 | 71 | 306 |
| Malignant neoplasms^b^ | 54 | 87 | 268 |
| All other causes | 177 | 182 | 631 |
|  |  |  |  |

^a^ ICD-9: 390-398, 402, 404, 410-434, 436-438; ICD-10: I00-I09, I11, I13, I20-I69.

^b^ ICD-9 14-208; ICD-10: C00-C97.
