# Supplementary material for: Predicting Survival from Telomere Length versus Conventional Predictors: A Multinational Population-Based Cohort Study
Source: PLoS One. 2016 Apr 6;11(4):e0152486. doi: 10.1371/journal.pone.0152486 (PMC4822878; doi:10.1371/journal.pone.0152486)
Supplement: S5 Table — CRELES (Costa Rica, N = 923, aged 61+), SEBAS (Taiwan, N = 976, aged 54+), and NHANES (U.S., N = 7822, aged 20+). (DOCX) [file pone.0152486.s014.docx]

S5 Table. Hazard Ratios (HR) and Gain in AUC Attributable to LTL and the Best Predictors^a^ of Cause-Specific Mortality Adjusted for Age and Sex. CRELES (Costa Rica, *N*=923, aged 61+), SEBAS (Taiwan, *N*=976, aged 54+), and NHANES (U.S., *N*=7822, aged 20+).

|  | **HR** | | |  | **Gain in AUC^c^** | | |
| --- | --- | --- | --- | --- | --- | --- | --- |
|  | **Costa**  **Rica** | **Taiwan** | **U.S.** |  | **Costa**  **Rica** | **Taiwan** | **U.S.** |
| **All-Cause Mortality** |  |  |  |  |  | | |
| a) LTL^b^ | 1.02 | 0.90+ | 0.91* |  | <0.001 | <0.001 | <0.001 |
| b) Self-Reported Mobility^b^ | 1.56*** | 1.36*** | 1.79***^,d^ |  | **0.024** | **0.015** | 0.035 |
| Mobility x Age | -- | -- | 0.996+ |  |  |  |  |
| c) Cognitive Function^b^ | 0.35**^,d^ | 0.75*** | ^e^ |  | 0.012 | 0.014 | ^e^ |
| Cognitive Function x Age | 1.01* | -- |  |  |  |  |  |
| d) Self-Assessed Health (SAH) | ^d^ |  | ^d^ |  | 0.006 | 0.008 | **0.037** |
| Poor (ref) | 1.00 | 1.00 | 1.00 |  |  |  |  |
| Fair | 0.04+ | 0.56* | 0.30** |  |  |  |  |
| Good | 0.01** | 0.54* | 0.19*** |  |  |  |  |
| Very Good | 0.03+ | 0.36** | 0.11*** |  |  |  |  |
| Excellent | 0.003* | 0.44** | 0.05*** |  |  |  |  |
| SAH x Age |  |  |  |  |  |  |  |
| Poor (ref) | 1.00 | -- | 1.00 |  |  |  |  |
| Fair | 1.05+ | -- | 1.01 |  |  |  |  |
| Good | 1.07* | -- | 1.01 |  |  |  |  |
| Very Good | 1.05+ | -- | 1.02* |  |  |  |  |
| Excellent | 1.09* | -- | 1.02* |  |  | | |
|  |  |  |  |  |  | | |
| **CVD Mortality** |  |  |  |  |  | | |
| a) LTL^b^ | 1.04 | 0.88 | 0.88+ |  | <0.001 | <0.001 | <0.001 |
| b) Self-Reported Mobility^b^ | 1.56** | 1.74*** | 1.63*** |  | **0.016** | **0.020** | 0.012 |
| c) SAH | ^d^ |  |  |  | 0.015 | 0.008 | **0.016** |
| Poor (ref) | 1.00 | 1.00 | 1.00 |  |  |  |  |
| Fair | 0.04* | 0.34* | 0.43*** |  |  |  |  |
| Good | 0.01** | 0.32* | 0.33*** |  |  |  |  |
| Very Good | 0.06 | 0.16** | 0.18*** |  |  |  |  |
| Excellent | 0.01* | 0.15** | 0.13*** |  |  |  |  |
| SAH x Age |  |  |  |  |  |  |  |
| Poor (ref) | 1.00 | -- | -- |  |  |  |  |
| Fair | 1.11+ | -- | -- |  |  |  |  |
| Good | 1.17** | -- | -- |  |  |  |  |
| Very Good | 1.08 | -- | -- |  |  |  |  |
| Excellent | 1.14+ | -- | -- |  |  |  |  |
| (continued on next page) |  |  |  |  |  |  |  |
| **Cancer Mortality** |  |  |  |  |  | | |
| a) LTL^b^ | 1.17 | 0.84 | 1.23+^d^ |  | 0.001 | 0.002 | 0.001 |
| LTL x Age | -- | -- | 0.994* |  |  |  |  |
| b) History of cancer | 4.25*** | 1.72 | 12.50***^,d^ |  | **0.019** | 0.004 | **0.012** |
| History of Cancer x Age | -- | -- | 0.97* |  |  |  |  |
| c) Smoking |  |  |  |  | 0.010 | 0.008 | **0.012** |
| Never smoked | 1.00 | 1.00 | 1.00 |  |  |  |  |
| Former smoker | 1.33 | 0.996 | 1.62** |  |  |  |  |
| Current smoker | 1.83 | 1.69+ | 2.97*** |  |  |  |  |
| d) Marital status |  |  |  |  | **0.027** | <0.001 | 0.003 |
| Married/partner | 1.00 | 1.00 | 1.00 |  |  |  |  |
| Widowed | 2.73** | 1.16 | 1.24 |  |  |  |  |
| Divorced/separated | 1.80 | 1.19 | 1.67 |  |  |  |  |
| Never married | 0.59 | 1.24 | 1.56 |  |  |  |  |
| e) Education |  |  |  |  | 0.017 | **0.014** | 0.002 |
| Very low | 1.00 | 1.00 | 1.00 |  |  |  |  |
| Low | 0.48 | 1.36 | 1.04 |  |  |  |  |
| Medium | 0.71 | 1.07 | 1.01 |  |  |  |  |
| High | 1.10 | 0.41+ | 0.97 |  |  |  |  |
| Very high | 1.07 | 0.78 | 0.60* |  |  |  |  |
|  |  |  |  |  |  |  |  |
| **Mortality From All Other Causes** | | | | | | | |
| a) LTL^b^ | 0.95 | 0.93 | 0.92 |  | <0.001 | <0.001 | <0.001 |
| b) Self-Reported Mobility^b^ | 1.80*** | 1.35*** | 1.50*** |  | **0.020** | 0.010 | **0.012** |
| c) SAH | ^d^ |  |  |  | 0.010 | 0.006 | **0.016** |
| Poor (ref) | 1.00 | 1.00 | 1.00 |  |  |  |  |
| Fair | 0.15 | 0.55+ | 0.42*** |  |  |  |  |
| Good | 0.07* | 0.48* | 0.32*** |  |  |  |  |
| Very Good | 0.07* | 0.33** | 0.24*** |  |  |  |  |
| Excellent | 0.002** | 0.41* | 0.15*** |  |  |  |  |
| SAH x Age |  |  |  |  |  |  |  |
| Poor (ref) | 1.00 | -- | -- |  |  |  |  |
| Fair | 1.06+ | -- | -- |  |  |  |  |
| Good | 1.07+ | -- | -- |  |  |  |  |
| Very Good | 1.07+ | -- | -- |  |  |  |  |
| Excellent | 1.17** | -- | -- |  |  |  |  |
| d) HbA1c^b^ | 1.19* | 1.42*** | 1.25*** |  | 0.006 | **0.020** | 0.006 |
|  |  |  |  |  |  |  |  |

+ *p* < 0.10, * *p* < 0.05, ** *p* < 0.01, *** *p* < 0.001, two-tailed.

^a^ For each outcome, we show the results for the top predictor in each country.

^b^ The HR represents the effect per SD of the specified predictor.

^c^ Change in the AUC is based on a comparison between a model that includes the specified predictor with one that excludes that predictor.

^d^ The effect of the predictor varied with age; the main effect represents the HR at age 20.

^e^ Cognitive function was not tested for NHANES because the assessment was administered only to respondents aged 60 and older.
